# Defects in DNA damage signaling and cell cycle checkpoints in a mouse model of *Rhno1* deletion

**DOI:** 10.1038/s41420-025-02912-z

**Published:** 2025-12-19

**Authors:** Joonyoung Her, Adithi Santhosh, Yanira Gonzalez-Rodriguez, Niphat Jirapongwattana, Channabasavaiah B. Gurumurthy, Adam R. Karpf, Samuel F. Bunting

**Affiliations:** 1 Department of Molecular Biology and Biochemistry, Rutgers, The State University of New Jersey, Piscataway, NJ USA; 2 Eppley Institute for Cancer Research and Fred & Pamela Buffett Cancer Center, University of Nebraska Medical Center, Omaha, NB USA; 3 Department of Cell and Molecular Biology, University of Mississippi Medical Center, Jackson, MS USA

**Keywords:** Cell growth, Mouse, Cancer genetics, DNA damage response, DNA recombination

## Abstract

In response to DNA damage or DNA replication stress, cells activate signaling pathways dependent on the kinase, ATR (Ataxia Telangiectasia and Rad3-Related). ATR signaling leads to induction of cell cycle checkpoints, a pause in DNA replication, and upregulation of DNA repair activities. In response to replication stress, ATR is activated by TOPBP1 (Topoisomerase II beta-Binding Protein 1) associated with the 9-1-1 (Rad9-Hus1-Rad1) complex. The three proteins that make up the 9-1-1 complex form a ring encircling DNA at damage sites and help localize TOPBP1 and ATR to signal the presence of damage or replication stress. RHNO1 (Rad9, Hus1, and Rad1-associated Nuclear Orphan 1) was identified as a protein that binds to components of the 9-1-1 complex to promote ATR signaling. Previous studies in cell lines have revealed that RHNO1 activity is required for maintenance of the G_2_M cell cycle checkpoint after ionizing radiation treatment, and for DNA repair in mitotic cells. In this study, we report a loss-of-function mouse model, in which *Rhno1* is deleted in B lymphocytes, allowing us to test the function of RHNO1 in primary cells. We find that RHNO1 is broadly expressed in mouse tissues but is dispensable for B cell growth under normal conditions. RHNO1-deficient B cells nevertheless show altered checkpoint responses and reduced ability to repair DNA damage in M phase. Whereas initial ATR activation after ionizing radiation treatment appears normal in RHNO1-deficient cells, ATR/CHK1 signaling is reduced at later timepoints. Joining of DNA breaks during class switch recombination, which is dependent on nonhomologous end-joining, is not significantly affected by loss of RHNO1. These results demonstrate that RHNO1, unlike other proteins required for ATR-CHK1 signaling, is not essential for growth of primary cells, but has specific roles in regulating responses to cell stress.

## Introduction

Cell cycle checkpoints become activated in response to DNA damage or replication stress to pause further growth, and provide an opportunity for recovery [1, 2]. The ATM (Ataxia Telangiectasia-Mutated) and ATR (Ataxia Telangiectasia-Related) kinases play a key role in the signal transduction pathways that lead to induction of cell cycle checkpoints. ATM is recruited by the heterotrimeric MRN (Mre11-Rad50-NBS1) complex to DNA double-strand breaks, leading to activation and phosphorylation of downstream mediators, including the checkpoint kinase, CHK2 [3]. ATR is recruited to stalled replication forks or DNA damage sites with its heterodimeric binding partner, ATRIP (ATR Interacting Protein) [4, 5]. Activation of ATR requires association with RPA (Replication Protein A) bound to single-stranded DNA, and the presence of either TOPBP1 (Topoisomerase II beta-Binding Protein 1) or ETAA1 (Ewing’s Tumor-Associated Antigen 1) [6–9]. Activated ATR phosphorylates a variety of downstream mediators, including the checkpoint kinase, CHK1 [10, 11]. In addition to induction of cell cycle checkpoints, ATR-CHK1 activity helps to stabilize replication forks, inhibit replication origin firing, and stimulate DNA repair.

The localization of TOPBP1 to sites of replication stress is dependent on the 9-1-1 complex (Rad9-Hus1-Rad1), which binds to TOPBP1 and facilitates the ATR-TOPBP1 interaction [11, 12]. The Rad9-Hus1-Rad1 Interacting Nuclear Orphan 1 (RHNO1) protein binds to the Rad1 and Rad9 components of the 9-1-1 complex and contributes to ATR activation [13–15]. The importance of RHNO1 was first revealed in a screen for factors required for a sustained G2/M checkpoint response after ionizing radiation (IR) treatment [13]. Depletion of *RHNO1* leads to defective ATR signaling and a premature re-entry into the cell cycle after IR treatment. RHNO1 has more recently been shown to have a role in DNA repair, specifically by recruiting DNA Polymerase θ (Pol θ) to DNA breaks to enable microhomology-mediated end joining (MMEJ) during mitosis [16]. As a player in both cell cycle checkpoint responses and DNA repair, RHNO1 is potentially a target for therapeutic approaches to treat cancer. This possibility is supported by multiple reports linking *RHNO1* expression to growth and survival of cancer cells [17–19].

To more clearly characterize the importance of RHNO1 in a living organism, we generated a novel *Rhno1* conditional-knockout mouse model. By deletion of *Rhno1* in the B lymphocyte lineage, we tested the requirements for RHNO1 for proliferation of a specific primary cell population. Conditional deletion in B lymphocytes also allows us to test the requirement for RHNO1 for nonhomologous end-joining in a physiological setting by quantifying the efficiency of class switch recombination [20]. We find that deletion of the *Rhno1* gene does not severely impact the growth of primary B cells, but leads to defects in ATR signaling at late timepoints after IR treatment. Defective ATR signaling correlates with increased chromosome instability, and a failure to properly induce cell cycle checkpoints after DNA damage. We also confirmed that RHNO1 contributes to DNA damage repair in mitosis. Finally, we show that RHNO1 is not required for non-homologous end joining-mediated repair of DNA double-strand breaks induced during class switch recombination.

## Results

### RHNO1 is broadly expressed in mouse tissues but is not essential for B cell development or proliferation

**Fig. 1:**
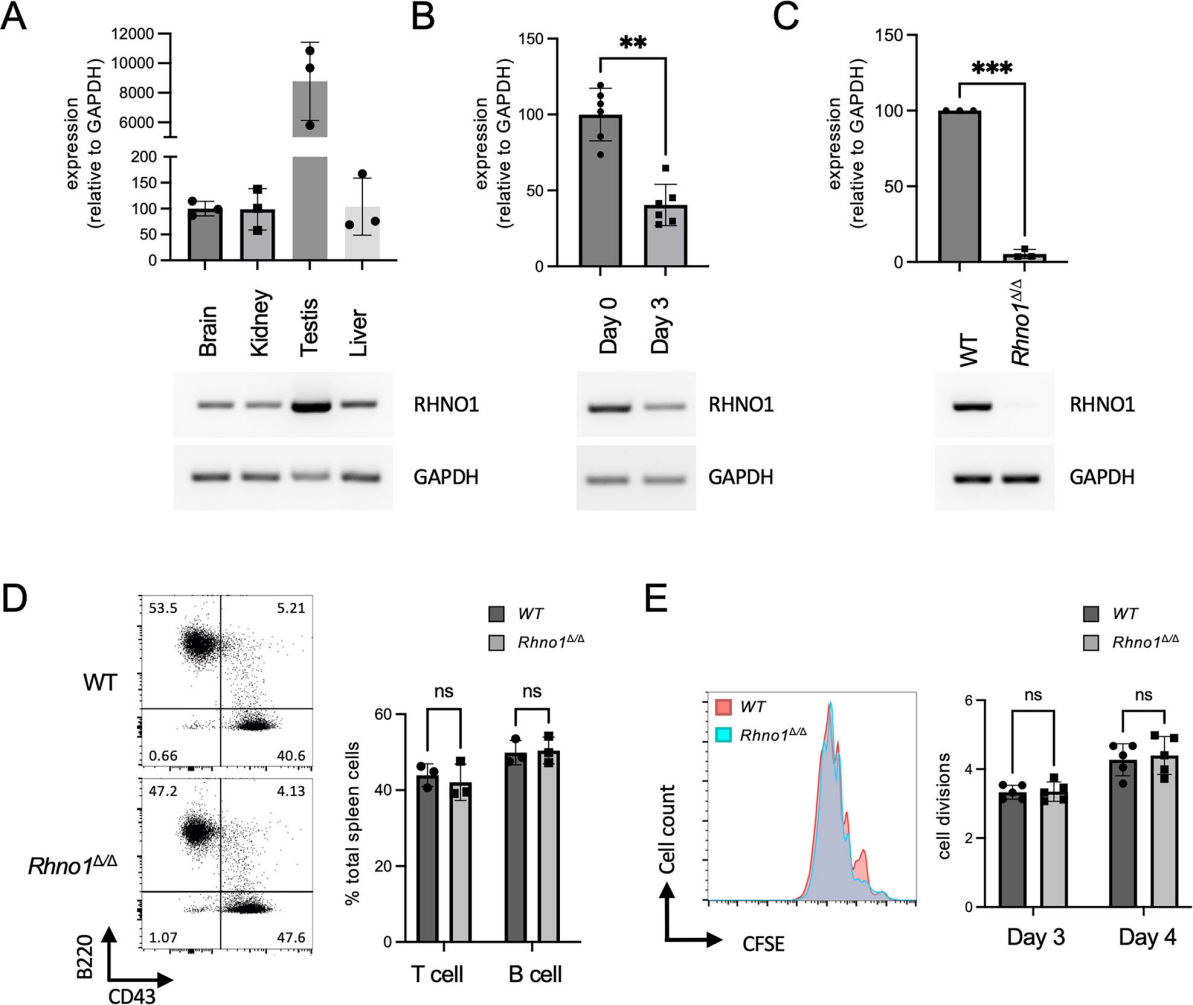
Quantification of *Rhno1* gene expression in WT and *Rhno1*^Δ/Δ^ cells. *Rhno1* mRNA expression in **A** freshly-isolated mouse tissues, **B** freshly-isolated (Day 0) and activated (Day 3) B lymphocytes, **C** Wildtype (WT) and *Rhno1*^Δ/Δ^ B lymphocytes, measured after 72 h in vitro culture. Charts show quantification by reverse-transcriptase quantitative PCR (RT-qPCR), normalized to *Gapdh*. A minimum of *n* = 3 samples were measured in triplicate in each case. Values in (**A**) are relative to brain. Values in (**B**) are relative to Day 0. Values in (**C**) are relative to WT. Accompanying gel images show results of semi-quantitative RT-PCR using the same primers and limited cycles. **D** Flow cytometry analysis of cell populations in the spleens of WT and *Rhno1* conditional-knockout mice. Figures show percentage of the total population of nucleated splenocytes staining for B220 and CD43. Graph shows mean proportion of total splenocytes that were T cells (B220^−^ CD43^+^) or B cells (B220^+^ CD43^-^). **E** Flow cytometry analysis of CFSE (carboxyfluorescein isothiocyanate) dilution to measure B cell proliferation in vitro. Representative flow cytometry data shows CFSE signal after 3 days in culture. Chart shows quantification of cell doublings based on CFSE fluorescence after 3 days or 4 days in culture. Error bars in parts (**A**–**E**) show standard deviation (S.D.) of the mean, with *P* values calculated by paired t-test.

The mouse *Rhno1* gene is expressed as a 235 amino acid protein with 68.8% identity to the 238-amino acid human ortholog (Fig S1A). As in humans, most of the mouse RHNO1 protein is predicted to be disordered (Fig S1B). To evaluate the expression of *Rhno1* in normal mice, we prepared RNA from multiple tissues and performed RT-qPCR (Fig. 1A). *Rhno1* expression was readily detectable in samples from brain, kidney, testis and liver, indicating that *Rhno1* is broadly expressed. We additionally prepared samples from freshly-isolated splenic B lymphocytes, which are in G0/G1 phase of the cell cycle, and from splenic B cells that had been induced to undergo active proliferation by treatment with lipopolysaccharide (LPS) (Fig. 1B) [21]. *Rhno1* expression was detected in resting B cells at levels equivalent to or higher than in activated B cells, indicating that *Rhno1* is expressed in both quiescent and dividing B lymphocytes.

We generated a conditional-knockout allele of *Rhno1* (*Rhno1*^fl^), in which LoxP sites were placed flanking the first coding exon, which contains the start site for translation (Fig S1C). Conditional deletion of *Rhno1* in the B cell lineage was achieved by crossing to a *CD19-Cre* knockin line [22]. *Rhno1* transcription was virtually undetectable in conditional-knockout *Rhno1*^Δ/Δ^ cells (Fig. 1C). Deletion of *Rhno1* did not affect the overall proportion of splenic B lymphocytes, however, nor did it significantly impact the ability of *Rhno1*^Δ/Δ^ B cells to proliferate in vitro (Fig. 1D, E). We conclude that expression of *Rhno1* is not essential for B cell differentiation or proliferation. We additionally derived *Rhno1*^−/−^ mice, with deletion of *Rhno1* in the whole body. Knockout mice were born at a frequency slightly below the expected Mendelian ratio (8 knockout mice born from a total of 56 pups, versus 14 expected). Importantly, *Rhno1*^−/−^ mice did not have major developmental or growth phenotypes, indicating that *Rhno1* expression is not essential for normal embryonic development.

### *Rhno1*^Δ/Δ^ B cells show altered DNA damage signaling at late timepoints after ionizing radiation treatment

**Fig. 2:**
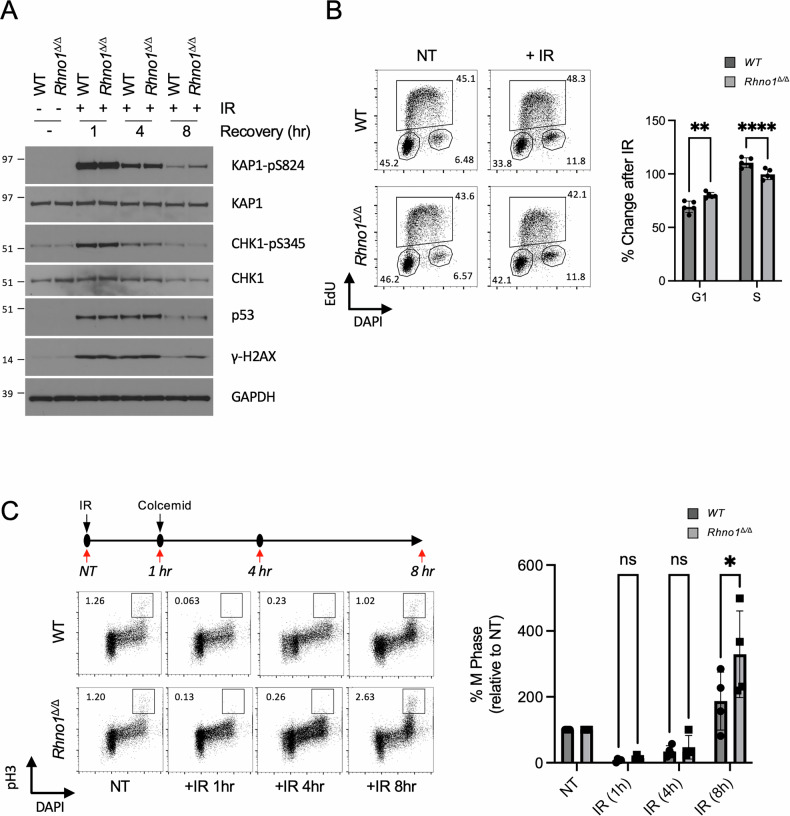
DNA damage signaling and cell growth after conditional knockout of *Rhno1.* **A** Western blot to show DNA damage signaling in WT and *Rhno1*^∆/∆^ cells after 5 Gy of ionizing radiation (IR) treatment at the indicated timepoints. **B** Flow cytometry analysis of cell cycle in non-treated (NT) cells and cells treated with IR (2 Gy, 3 h recovery). Chart shows the percentage change of G1 and S phase cell populations compared to non-treated controls. **C** Flow cytometry analysis of mitotic (pH3^+^) cells after 5 Gy of IR treatment, with recovery for the times shown. Chart shows the percentage change of the mitotic cell population compared to untreated cells. Error bars in (**B**) and (**C**) show S.D. of the mean, with P values calculated by paired t-test.

RHNO1 has been reported to be involved in DNA damage signaling and control of cell cycle checkpoints [13, 15, 17]. To test this in primary cells, we treated WT and *Rhno1*^Δ/Δ^ splenic B cells with ionizing radiation (IR) to induce DNA double-strand breaks. One hour after treatment, both WT and *Rhno1*^Δ/Δ^ cells showed equivalent levels of phosphorylation of the ATM substrate, KAP1, and the ATR substrate, CHK1 (Fig. 2A, Fig S3A). Stabilization of p53 was also observed in both WT and *Rhno1*^Δ/Δ^ cells at early timepoints after irradiation. To test the effect of RHNO1 deletion in the cellular responses to other forms of DNA damage, we measured levels of pKAP1 and pCHK1 after treatment with hydroxyurea, camptothecin, or olaparib, which inhibit ribonucleotide reductase, topoisomerase I and Poly(ADP-Ribose) Polymerase, respectively. All of these treatments caused induction of pKAP1 and pCHK1, but no differences were observed between the WT and *Rhno1*^Δ/Δ^ groups (Fig S2A, B, Fig S3B). The levels of KAP1 and CHK1 phosphorylation diminished at 4 h post-irradiation but no significant differences between WT and conditional-knockout cells were observed at this timepoint. *Rhno1*^Δ/Δ^ cells showed a higher level of KAP1 phosphorylation at 8 h post-irradiation, however, indicating ongoing ATM-dependent DNA damage signaling. At 8 h post-irradiation, there was also a lower level of CHK1 phosphorylation in *Rhno1*^Δ/Δ^ cells, consistent with earlier reports demonstrating a requirement for RHNO1 in ATR/CHK1 signaling [13, 15]. We conclude that RHNO1 is not essential for the initial activation of ATR in response to these treatments, but contributes to sustained signaling at later timepoints.

We next tested whether the altered DNA damage signaling in *Rhno1*^Δ/Δ^ cells produced changes in cell growth and cell cycle checkpoints after IR treatment. Untreated *Rhno1*^Δ/Δ^ cells showed equivalent numbers of cells in each phase of the cell cycle as WT controls (Fig. S4A), but the pattern was altered after IR treatment (Fig. 2B). Notably, *Rhno1*^Δ/Δ^ cells showed a lower proportion of cells in S phase and an increased proportion of cells in G1 after IR treatment. By measurement of early and late S phase populations, we found that *Rhno1*^Δ/Δ^ cells did not show a difference in S phase progression at early timepoints after IR, but appeared to complete the cell cycle and return to G1 more quickly than WT cells (Fig S4B). RHNO1 was reported to be required for maintenance of the IR-induced G2/M cell cycle checkpoint [13]. To test this, we quantified the proportion of mitotic cells at various timepoints after IR treatment, using a colcemid trap assay to prevent progression of cells into G1 phase (Fig. 2C). *Rhno1*^Δ/Δ^ cells showed a very low proportion of mitotic cells at the 1-hour timepoint after IR, indicating that the G2/M checkpoint is initially induced normally in these cells. However, at the 8-h timepoint, *Rhno1*^Δ/Δ^ cells showed a significantly elevated proportion of mitotic cells compared to WT controls, consistent with a defect in the maintenance of G2/M arrest after IR treatment.

### Chromosome instability and altered cell growth in *Rhno1*^Δ/Δ^ cells

**Fig. 3:**
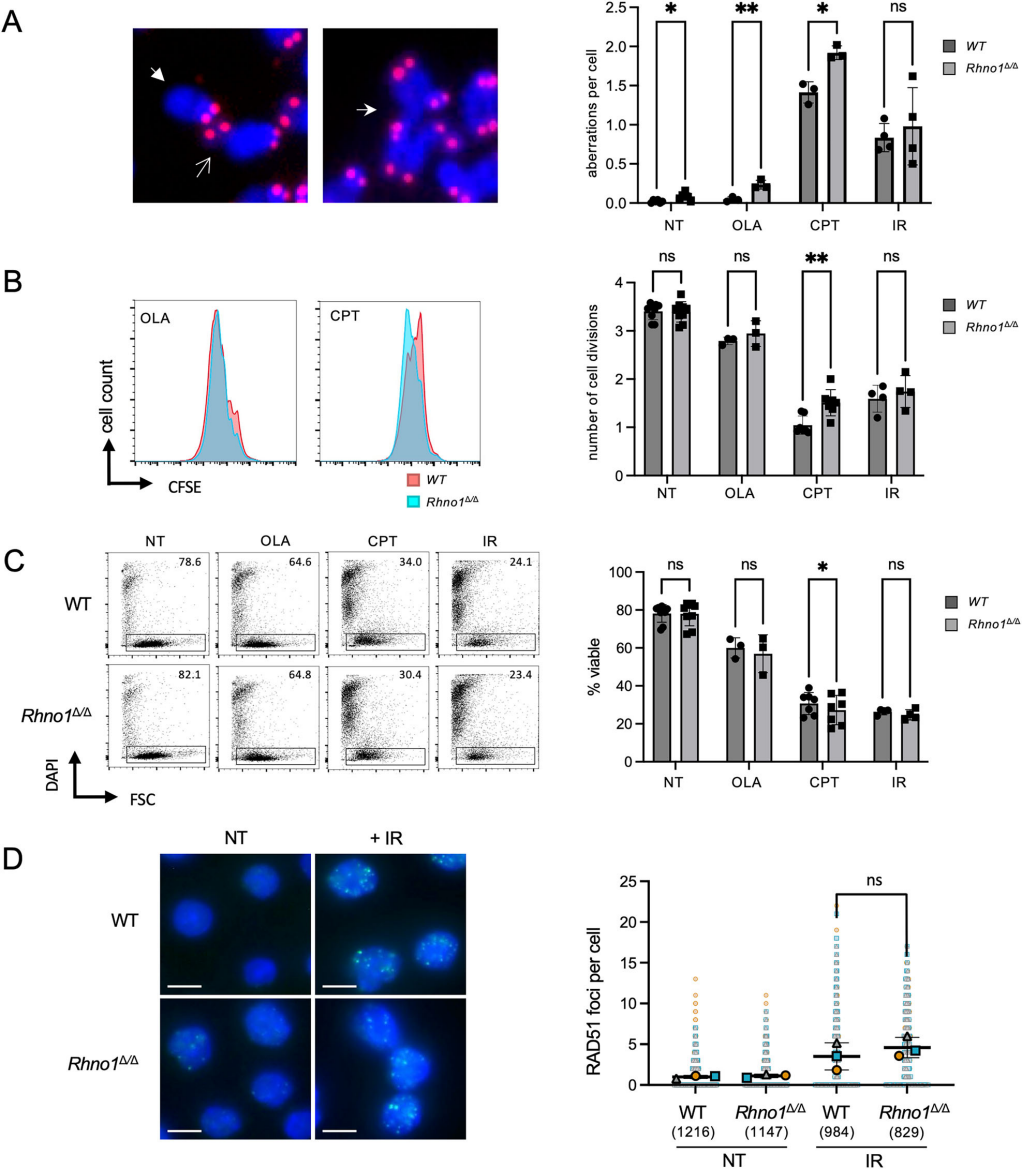
Responses to genotoxic stress in *Rhno1*^Δ/Δ^ cells*.* (**A**) Analysis of chromosome aberrations in metaphase spreads prepared from WT and *Rhno1*^∆/∆^ B cells that were non-treated (NT) or treated with 2 μM Olaparib (OLA), 50 nM camptothecin (CPT), or 2 Gy of ionizing radiation (IR). Chromosome preparations were fixed 16 h after treatment in each case. Solid arrowhead shows a chromosome break, open arrowhead shows a chromatid break, and stealth arrowhead shows a radial chromosome. **B** Flow cytometry analysis of CFSE dilution to measure B cell growth after 72 h treatment with 1 μM OLA or 50 nM CPT, or IR. IR treatment (5 Gy) was administered 24 h after activation, with analysis 48 h later. Chart shows quantification of cell doublings based on CFSE fluorescence. **C** Analysis of viability of *Rhno1*^∆/∆^ cells 72 h after activation. Treatments were as **B**. **D** Immunofluorescent quantification of RAD51 foci in cells after no treatment (NT), or after 4 h recovery from treatment with 10 Gy of IR. Scale bar: 5 μm. Data points in graph show values from *n* = 3 independent experiments. Data points from each experiment are represented with a different symbol and color. The total number of cells analyzed in each group is indicated in parentheses. A minimum of 829 cells was analyzed in each group. Error bars show the S.D. of the mean of the means of three independent experiments. *P* values were calculated with paired t-test. ns not significant.

To evaluate the importance of RHNO1 for DNA repair responses, WT and *Rhno1*^Δ/Δ^ B cells were activated by LPS in vitro and treated either with vehicle, or one of three treatments to induce DNA damage. In particular, we used Olaparib, a poly(ADP-ribose) polymerase (PARP) inhibitor, which increases the frequency of DNA double-strand breaks (DSBs) by interfering with DNA damage signaling [23]. We also used camptothecin, an inhibitor of topoisomerase I, which produces DSBs in S phase cells [24]. These treatments were compared to IR treatment, which generates DSBs and a mixture of other types of DNA damage during all phases of the cell cycle. Untreated *Rhno1*^Δ/Δ^ cells showed a small but significant increase in chromosome aberrations compared to WT cells (Fig. 3A). Notably, the frequency of chromosome aberrations was also higher in *Rhno1*^Δ/Δ^ cells as compared to WT cells after either Olaparib or camptothecin treatment. IR treatment caused an increase in the frequency of chromosome aberrations in both WT and *Rhno1*^Δ/Δ^ cells, but there was no significant difference between the control and knockout groups.

The impact of chromosome instability on cell growth was measured using a CFSE (Carboxyfluorescein Succinimidyl Ester) dilution assay (Fig. 3B). Each of the treatments inhibited the proliferation of both WT and *Rhno1*^Δ/Δ^ cells, but *Rhno1*^Δ/Δ^ cells proliferated more than WT cells after camptothecin treatment, even though they accumulated more chromosome aberrations under these conditions. We also measured the amount of cell death caused by Olaparib, camptothecin, or IR treatment (Fig. 3C). Although each of these treatments had a cytotoxic effect in both WT and *Rhno1*^Δ/Δ^ cells, there was no difference in the amount of cell death between the groups after either Olaparib or IR treatment. On the other hand, *Rhno1*^Δ/Δ^ cells treated with camptothecin showed greater cell death than WT controls. These results suggest that a failure to properly signal camptothecin-mediated DNA damage leads to uncontrolled proliferation, increased genomic instability, and cell death in the absence of RHNO1.

One possible pathway for repair of DSBs is homology-dependent repair (HDR) [25]. Evidence in other systems has indicated that RHNO1 may promote HDR [13, 17]. To test the requirement for RHNO1 in HDR in primary B cells, we quantified nuclear foci of RAD51, which form at DSBs induced by IR (Fig. 3D) [26]. We found that *Rhno1*^Δ/Δ^ cells formed RAD51 foci normally after IR treatment, indicating that RHNO1 is not essential for HDR in B cells.

### RHNO1 contributes to mitotic DNA repair in B cells

**Fig. 4:**
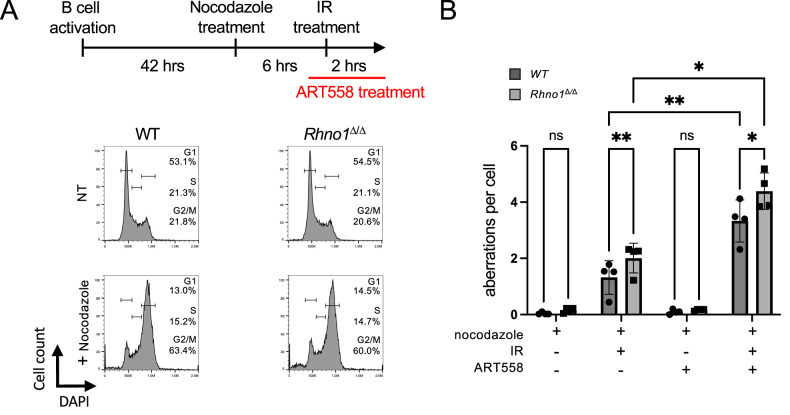
Genomic instability after ionizing radiation treatment in mitotic *Rhno1*^Δ/Δ^ cells. **A** Diagram of experimental design and representative flow cytometry data to validate mitotic arrest of B cells by nocodazole treatment. Cells were treated with nocodazole, and subsequently the Pol θ inhibitor, ART558. **B** Analysis of chromosome aberrations in metaphase spreads prepared from WT and *Rhno1*^∆/∆^ B cells 2 h after 2 Gy IR treatment under nocodazole-induced mitotic arrest. Error bars in (**B**) show the S.D. of the mean. P values were calculated by two-way ANOVA with Tukey’s multiple comparison test.

RHNO1 is reported to act during mitosis to mediate Pol θ-dependent repair of DSBs by MMEJ [16]. To test for a role for RHNO1 in mitotic MMEJ in primary cells, we induced WT and *Rhno1*^Δ/Δ^ splenic B lymphocytes to proliferate in vitro, and then treated with nocodazole, which disrupts the mitotic spindle and causes cells to arrest in mitosis (Fig. 4A). Cells trapped at mitosis by nocodazole treatment were exposed to IR, and chromosome aberrations were scored. Although asynchronously-dividing *Rhno1*^Δ/Δ^ cells did not show an increase in chromosome instability after IR treatment (Fig. 3A), we observed an increase in chromosome aberrations in *Rhno1*^Δ/Δ^ cells relative to WT controls after trapping the cells in mitosis (Fig. 4B). This result is consistent with a specialized role for RHNO1 in DNA repair during mitosis. To further test the involvement of Pol θ in mitotic DNA repair in *Rhno1*^Δ/Δ^ cells, we repeated the chromosome instability assay with pre-treatment with the Pol θ inhibitor, ART558. Treatment with ART558 caused a significant increase in chromosome aberrations in mitotic B cells that were exposed to IR. However, *Rhno1*^Δ/Δ^ cells showed a higher rate of chromosome aberrations after IR, even in the context of Pol θ inhibition. This result suggests that RHNO1 may contribute to mitotic DNA repair by a Pol θ-independent pathway in *Rhno1*^Δ/Δ^ B cells.

### RHNO1 is not required for microhomology-mediated repair during class switch recombination

**Fig. 5:**
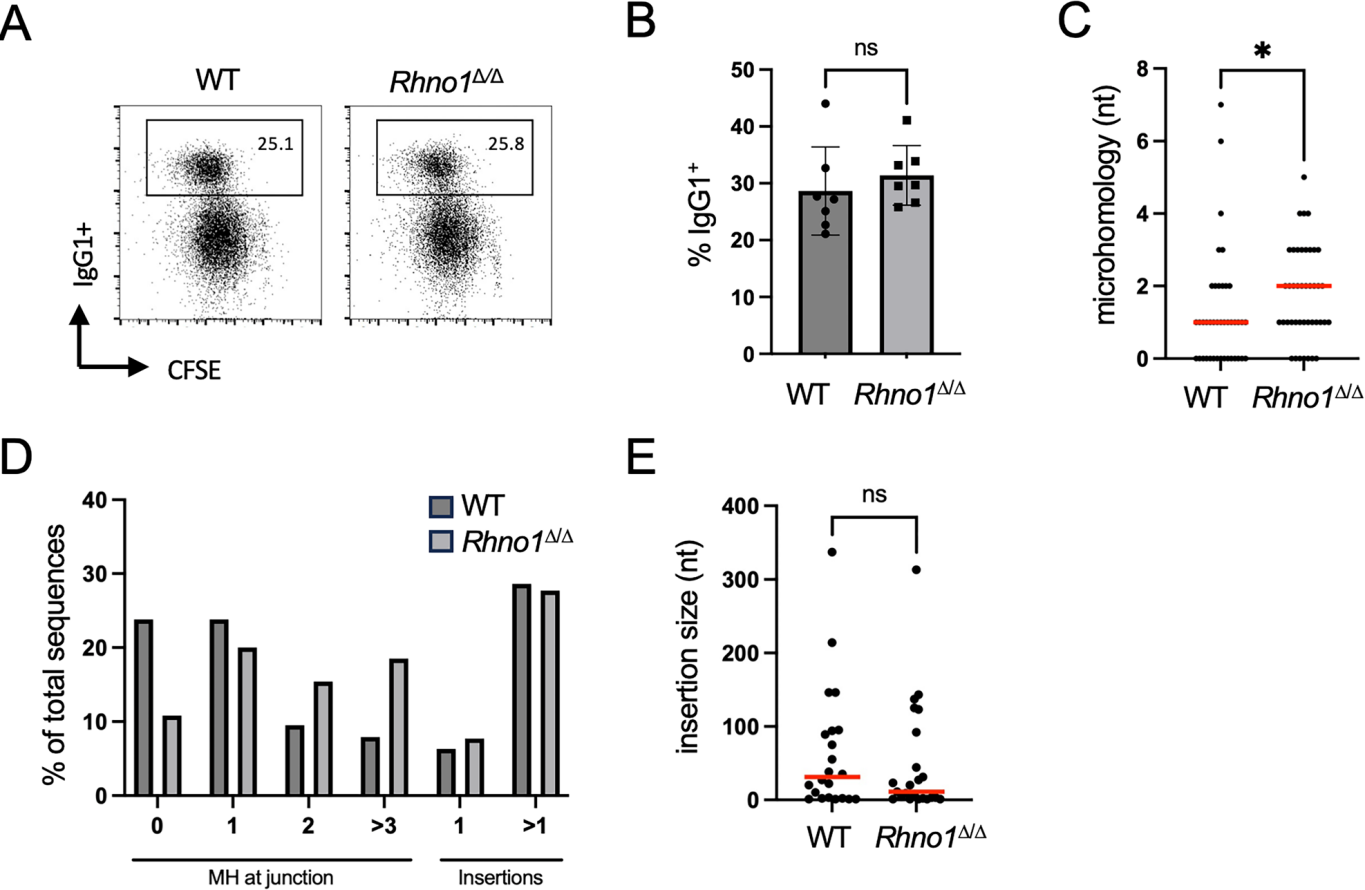
Class switch recombination in *Rhno1*^Δ/Δ^ cells. **A** Representative flow cytometry data of B cells after 96 h in vitro culture with lipopolysaccharide (LPS) and interleukin-4 (IL-4). **B** Quantification of IgG1^+^ populations in live WT and *Rhno1*^∆/∆^ B cells. **C** Microhomology use at class switch recombination junctions cloned from individual WT (*n* = 41) and *Rhno1*^∆/∆^ (*n* = 42) B cells. Red bars indicate median values. **D** Nucleotides of microhomology (MH) or insertions at class switch recombination junctions. **E** Size of insertions present at class switch recombination junctions from WT and *Rhno1*^∆/∆^ cells. Red bars indicate median values. Error bars in (**B**) represent S.D. of the mean. *P* values were calculated by two-tailed paired t-test. *P* values in (**C**) and (**E**) calculated by Mann-Whitney test.

Activated B lymphocytes undergo class switch recombination to modify the isotype of immunoglobulin that they produce [27]. Class switch recombination involves Activation-Induced Cytidine Deaminase (AID)-dependent production of DNA double-strand breaks at the immunoglobulin heavy chain locus, followed by repair by both the classical and microhomology-mediated end joining pathways. We measured the efficiency of class switch recombination to test the activity of these pathways in WT and *Rhno1*^Δ/Δ^ B cells (Fig. 5A, B). Notably, the frequency of class switch recombination to IgG1 was equivalent in *Rhno1*^Δ/Δ^ cells and WT controls. Moreover, the extent of microhomology present at class switch recombination junctions in *Rhno1*^Δ/Δ^ B cells was equal to or greater than that seen in WT B cells (Fig. 5C). The frequency and size of insertions at repair junctions were, likewise, similar in WT and *Rhno1*^Δ/Δ^ cells. We conclude that RHNO1 is not required for repair of DNA double-strand breaks formed during class switch recombination in B cells, and is not necessary for MMEJ in this context.

## Discussion

RHNO1 was first identified as a transcript that is expressed at high levels in breast cancer cells [19]. Overexpression of RHNO1 has since been documented in numerous types of cancer, and studies with RHNO1-deficient cells have supported the idea that RHNO1 activity is important for the growth of cancer cells [17, 18, 28]. These observations underscore the importance of understanding the cellular activities of RHNO1, and determining whether inhibition of RHNO1 might be a useful approach for the selective targeting of cancer cells. Our study represents the first attempt to test the effect of loss of RHNO1 in primary mammalian cells. Whereas we have so far been unable to identify an antibody that is specific for endogenous RHNO1, we confirmed that, as expected, the expression of *Rhno1* mRNA is virtually undetectable in conditional-knockout *Rhno1*^Δ/Δ^ B cells. *Rhno1*^Δ/Δ^ cells also show a clear defect in maintenance of the G_2_M cell cycle checkpoint after exposure to ionizing radiation, consistent with previous knockdown studies [13]. In contrast, *Rhno1*^Δ/Δ^ cells do not show a defect in ATR-CHK1 signaling at early timepoints after either ionizing radiation treatment, or after treatment with hydroxyurea, camptothecin, or Olaparib. RHNO1 therefore appears to be dispensable for the initial activation of ATR, at least in response to these treatments. RHNO1 instead appears to contribute to sustained ATR signaling, as shown by our observation of diminished CHK1 activation in *Rhno1*^Δ/Δ^ cells at late timepoints after recovery from IR treatment. *Rhno1*^Δ/Δ^ cells also showed a failure to properly arrest cell growth, especially after camptothecin treatment, which correlated with an increased number of metaphase chromosome aberrations and reduced cell viability.

The relatively mild impact of *Rhno1* deletion on ATR-CHK1 activation in primary B cells is consistent with the observed effect of whole-body deletion of *Rhno1*. We found that *Rhno1*^−/−^ mice, in which *Rhno1* is deleted in all tissues, are viable, and show no obvious developmental defects. In contrast, whole-body deletion of other genes required for ATR-CHK1 signaling causes profound developmental defects. *Atr*^−/−^ mice and *Chk1*^−/−^ *mice* show embryonic lethality at an early stage of gestation [29–31]. Deletion of *Topbp1*, or any of the three components of the 9-1-1 complex, also results in early embryonic lethality [32–35]. Even reduced expression of ATR, as is seen in *Atr*^*Seckel*^ mice, causes stunted growth, microcephaly, and severely reduced lifespan [36]. *Rhno1*^−/−^ mice appear more similar to *Etaa1*^−/−^ mice, which are viable, although are born at a sub-Mendelian ratio [6,37,38]. ETAA1 is known to have a specific role in sustaining ATR activity during normal cell growth but is less important for responses to DNA damage or acute replication stress [6, 37, 39]. Based on the relatively mild impact of *Rhno1* deletion on normal cells, it is likely that potential therapies to target RHNO1 activity will have limited toxicity in non-cancerous tissues. Preclinical studies have demonstrated that depletion of RHNO1 strongly inhibits the growth of cancer cells [28]. This effect on cancer cells contrasts with our results with primary B cells, in which deletion of *Rhno1* did not significantly affect cell growth under unperturbed conditions. CRISPR-mediated deletion of *RHNO1* likewise did not significantly affect the growth of a cell line derived from normal fallopian tube epithelial cells [17].

The proteins CLOCK, INTS7, MGC13204, BRCA2, and PALB2 are also required to prevent premature re-entry into the cell cycle after IR treatment [13, 40]. The mechanism by which these proteins regulate checkpoint maintenance is not fully characterized. Evidence for how RHNO1 sustains a prolonged G_2_M checkpoint is based on biochemical and structural biological studies, which have shown that RHNO1 can interact with TOPBP1 and the RAD1 and RAD9 components of the 9-1-1 complex [13, 15, 41, 42]. RHNO1 binds to RAD1 at a site that overlaps the binding interface for the 9-1-1 clamp loader, RAD17-RFC [43]. Expression of RHNO1 may therefore stabilize the 9-1-1 complex on DNA by preventing premature unloading mediated by RAD17-RFC. Alternatively, RHNO1 may ensure sustained activity of the 9-1-1 complex by preventing an inhibitory effect caused by intra-molecular binding of the RAD9 tail domain [14]. Our results are consistent with either of these models for RHNO1-mediated upregulation of ATR activation, which prolongs the G_2_M checkpoint.

Several reports have indicated that RHNO1 is required for homology-dependent repair (HDR) [13, 17]. Genetic deficiencies that compromise HDR lead to defects in cell proliferation, reduced formation of nuclear RAD51 foci, and hypersensitivity to PARP inhibitors [44–48]. We did not observe these phenotypes in *Rhno1*^Δ/Δ^ cells; therefore RHNO1 does not appear to be essential for HDR in B cells. The difference with previous studies may reflect altered requirements for HDR in different cell types. Previous studies have used cell lines adapted to long-term in vitro culture, whereas our study uses primary cells taken directly from mouse spleen. Cancer cell lines may grow under heightened levels of replication stress, or with defects in factors such as p53, which regulate normal responses to DNA damage. A key role for RHNO1 in microhomology-mediated end-joining (MMEJ) was identified in cells lacking normal HDR and classical NHEJ activities [13]. This study revealed that RHNO1 has a specific role in in recruiting Pol θ to mediate MMEJ in mitotic cells. Our results with cells trapped in mitosis are consistent with a role for RHNO1 in end-joining, which is either distinct from or parallel to any role in ATR/CHK1 signaling. We observed that mitotic cells lacking RHNO1 showed an increase in chromosome aberrations after IR treatment, which likely reflects a defect in DNA repair. We also found that RHNO1-deficient cells had a higher frequency of chromosome aberrations in the context of Pol θ inhibitor, potentially suggesting that RHNO1 may contribute to DNA repair in mitotic cells by mechanisms that are independent of Pol θ.

The efficiency of immunoglobulin gene rearrangements in B lymphocytes that lack RHNO1 gives insight into the requirements for RHNO1 in specific DNA repair pathways. V(D)J recombination produces mature immunoglobulin genes by RAG1/2-mediated induction of DNA double-strand breaks, which are repaired by the ‘classical’ or ‘canonical’ nonhomologous end-joining pathway (C-NHEJ) [49]. Knockout mice that lack components of the C-NHEJ pathway, such as Ku80 or DNA Ligase IV, cannot complete V(D)J recombination and show a complete loss of mature lymphocytes [50–52]. The presence of normal B cell populations in *Rhno1* conditional-knockout spleens indicates that V(D)J recombination takes place normally in these mice, and RHNO1 is therefore not required for C-NHEJ. Mature B cells can undergo a second process of rearrangement of the immunoglobulin heavy chain loci, called class switch recombination (CSR) [49], which also proceeds through induction and repair of DNA double-strand breaks [20]. Whereas V(D)J recombination is almost always dependent on C-NHEJ, DNA double-strand breaks formed during CSR can also be repaired by MMEJ [53–55]. Microhomology was detectable at the repair junctions of *Rhno1*^Δ/Δ^ B cells at a rate that was equivalent to, or higher, than that observed in WT B cells; therefore RHNO1 does not appear to be necessary for MMEJ during CSR. This result does not preclude the possibility that RHNO1 may contribute to MMEJ in other contexts. Notably, Pol θ-knockout mice are also competent for CSR [56], although Pol θ is clearly required for MMEJ in other assays [57].

In conclusion, we find that loss of *Rhno1* does not severely compromise the growth of primary B cells under normal growth conditions, but RHNO1 contributes to ATR signaling and DNA repair when cells are challenged by replication stress, or when pathways for DNA repair are deficient [16]. As these conditions exist in a range of cancer cases, inhibition of RHNO1 activity may be a useful approach for targeted anti-cancer therapies. Future studies should further test the impact of loss of RHNO1 activity in tumor models, to evaluate whether targeting RHNO1 is likely to have clinical benefit.

## Materials and methods

### Generation of Rhno1 conditional knockout mice

A floxed allele for the mouse *Rhno1* gene (ENSMUSG00000048668) was produced by targeting exon 3 using the Easi-CRISPR method [58, 59]. The upstream and downstream LoxP sites were placed 73 bases and 225 bases away from exon 3, respectively. The single-stranded DNA donor, guide RNAs, and Cas9 protein used in the Easi-CRISPR method were procured from IDT, Coralville, Iowa. The C57BL6/J mouse strain (Jackson Labs, stock number 000664) was used as zygote donors to create the mouse model. Mouse zygote production, microinjection, animal husbandry and mouse genotyping protocols are as described [58, 60]. *Rhno1*^+/-^ mice were derived by crossing *Rhno1*^fl/+^ mice to EIIA-Cre mice (Jackson Labs, stock number 003724). Both male and female mice were used. All mouse work was carried out in conformity with a protocol approved by the Rutgers University Institutional Animal Care and Use Committee.

### Cell culture

Primary B cells were isolated from mouse spleen by depletion with CD43 MACS beads (130-049-801; Miltenyi), and activated for in vitro culture with lipopolysaccharide (LPS, L2630; Sigma) and interleukin 4 (404-ML/CF; R&D Systems) as previously described [61]. To test DNA repair efficiency during M phase, mitotic B cells were enriched by treatment with 100 ng/ml nocodazole (M1404; Sigma) for 6 h and subsequently treated with 2 Gy ionizing radiation from a ^137^Cs source. After 2 h recovery, cells were harvested to make metaphase spreads.

### Antibodies and chemicals

Commercial antibodies used in this study include: B220-Alexa 647 (557683; BD), CD43-PE (12-043-82; Invitrogen), p53 (2524; CST), KAP1-pS824 (A300-767A; Bethyl), KAP1 (A300-274A; Bethyl), CHK1-pS345 (2341; CST), CHK1 (sc-8408; Santa Cruz), γ-H2AX (05-636; Millipore), γ-H2AX-Alexa 488 (20304S; CST), Histone H3-pS10 (06570; Millipore), GAPDH (MAB374; Sigma), RAD51 (sc-8349; Santa Cruz), IgG1-biotin antibody (553441; BD), anti-CD16/CD32 (553142; BD). The following chemicals were used: hydroxyurea (H8627; Sigma), Olaparib (KU0059436; Selleckchem), camptothecin (C9911; Sigma), ART558 (S9936; Selleckchem), colcemid (10295892001; Sigma), and CFSE (21888; Sigma).

### Cell treatments and flow cytometry

To measure class switch recombination, purified B cells were labeled with 5 μM CFSE for 10 min at 37 °C, and then cultured at a density of 2.5 × 10^5^ cells/ml with or without IL-4 and LPS for 96 h. B cells were resuspended and incubated with anti-CD16/CD32 for 10 min at room temperature, followed by incubation with IgG1-biotin antibody for 1 h at 4 °C. After washing, Streptavidin-Alexa 647 (S32357; Invitrogen) was used for secondary labeling, and DAPI (4’,6-diamidino-2-phenylindole) exclusion was performed to identify dead cells. To test G_2_M checkpoint induction and recovery, B cells cultured in vitro for 48 h were exposed to 5 Gy ionizing radiation. 1 h after IR treatment, 10 ng/ml colcemid was added to block mitotic exit. Cells harvested at the indicated timepoints were fixed with ice-cold methanol for 20 min at −20 °C, then stained with anti-Histone H3-pS10 and DAPI. To measure S-phase progression after IR, Day 2 B cells were pulsed with 30 μM EdU (900584; Sigma) for 30 min to label the S-phase population. After EdU washout, cells were treated with 5 Gy ionizing radiation and then harvested at the indicated timepoints. The proportions of cells in each phase of the cell cycle were determined by EdU detection using the Click-iT EdU Alexa Fluor-647 imaging kit (C10340; Invitrogen), and DAPI staining to reveal DNA content. For the CFSE cell proliferation assay, purified B cells were resuspended at 0.5 × 10^6^ cells/ml in RPMI medium, labeled with 5 μM CFSE for 10 min at 37 °C, and cultured with or without IL-4 and LPS for 72 h. The CFSE fluorescence of unstimulated cells was set at 100%, and the number of cell divisions was calculated using log_2_(median intensity of unstimulated cells/median intensity of indicated cells). For EdU incorporation, B cells were pulsed with 30 μM EdU for 30 min, fixed with ice-cold methanol for 20 min, and EdU was detected using the Click-iT EdU Alexa Fluor-647 imaging kit. DAPI staining was used to measure DNA content [62]. Flow cytometry was performed using a Cytek Aurora, with analysis in FlowJo.

### Preparation of metaphase spreads and FISH

Fluorescent In Situ Hybridization (FISH) to label telomeres was performed as previously described [61]. B cells were first activated for 24 h with LPS and IL-4, and then treated for 16 h with the indicated reagents. Cells were arrested in metaphase by treatment with 100 ng/ml colcemid for 1 h. After harvesting, the cells were resuspended in a hypotonic solution (0.075 M KCl) for 15 min at 37 °C and then fixed using a 3:1 v/v mixture of methanol and acetic acid. The fixed cells were stored overnight at −20 °C. The fixed cell suspensions were dropped onto glass microscope slides in a Thermotron CDS-5 environmental chamber set to 22.9 °C and 52% humidity. These slides were dried for 30–60 min and subsequently stored in a 37 °C chamber. For FISH, a probe mix containing Cy3-labeled peptide nucleic acid probe complementary to mouse telomeric DNA repeats (Cy3-00-CCCTAA CCCTAACCCTAA, F1002; PNA Bio Inc.), was incubated at 37 °C for 1 h in deionized formamide (pH 7.0), followed by addition of a solution of 4× SSC, 20% dextran sulfate and incubation for an additional 1 h at 37 °C. The probe was then denatured at 80 °C and incubated for 1 h at 37 °C. Chromosome slides were prepared by incubation for 90 s at 37°C with pepsin (4 μg/ml, P6887; Sigma) in a 0.01 M HCl solution. Slides were then washed first in 1x PBS and next in 1x PBS/50 mM MgCl_2_. The slides were subsequently fixed for 10 minutes using 1% formaldehyde/1x PBS/50 mM MgCl_2_. After fixation, the slides were washed in 1x PBS and dehydrated through an ethanol series (70%, 90%, and 100%), then air-dried. The slides were denatured on a hot plate by incubating at 80 °C for 90 s in a 70% deionized formamide/2x SSC solution. This was immediately followed by another ethanol dehydration series (70%, 90%, and 100%) and air-drying. The pre-annealed probe mixture was applied to the prepared slides in a humid chamber, and the slides were incubated at 37 °C for 1 h, covered with a coverslip. The slides subsequently underwent three 5 min washes in 50% formamide/2x SSC, 1x SSC, and finally 4x SSC/0.1% Tween-20. The slides were stained with DAPI solution (80 ng/ml) and mounted with Mowiol antifade solution (81381; Sigma). Images were acquired with an AxioImager.Z2 microscope (Zeiss) using a 60× objective with MetaSystems automatic stage. For each experiment, a minimum of 50 metaphases per group were analyzed.

### Reverse transcription quantitative PCR

Total RNA was purified using the RNeasy mini kit (74106; Qiagen) and reverse transcribed with SuperScript^TM^ III Reverse Transcriptase (18080; Invitrogen) and random hexamers (N8080127, Invitrogen). cDNA samples were amplified and measured in triplicate with PowerUp^TM^ SYBR^TM^ Green Master Mix (A25742; Applied Biosystems) using QuantStudio^TM^ (ThermoFisher). All expression levels were normalized to GAPDH. The following primers were used: mRHNO1 For (5’-CCAAACACCACTATGAATCTTGC-3’), mRHNO1-1 Rev (5’-GTCTCTGAACGGAAGACTGTG-3’), mGAPDH For (5’-GTTGTCTCCTGCGACTTCA-3’), mGAPDH Rev (5’-GGTGGTCCAGGGTTTCTTA-3’). Representative gel images were obtained using equivalent RT-PCR products produced using the primers as listed above with Hot Start Taq 2x Master Mix (M0496; NEB) under unsaturated conditions.

### Class switch recombination junction analysis

For cloning of repair junctions formed at immunoglobulin switch regions during class switch recombination, we adapted the strategy as described [63, 64]. The forward primer, annealing at Sμ, had the sequence 5’-TGGCTTAACCGAGATGAGCC-3’. The reverse primer, annealing at Sγ1, had the sequence 5’-CAATTAGCTCCTGCTCTTCTGTGG-3’. Genomic DNA was isolated from B lymphocytes cultured for 72 h in vitro with LPS and IL-4 [61]. An equimolar mix of DNA from three WT mice or three *Rhno1*^fl/fl^; CD19-Cre conditional knockout mice was used for PCR. PCR amplification used Pfu Turbo polymerase (Stratagene), with subsequent A-tailing (72 °C, 10 min) using GreenTaq (Genscript). Products were cloned into the TOPO-TA cloning vector (Thermo Fisher) and sequenced by Sanger sequencing (Azenta Genewiz). Sequence files were analyzed and annotated in Snapgene (GSL Biotec). A total of 63 unique sequences were identified from WT B cells, and compared to 65 unique sequences identified from *Rhno1* conditional-knockout B cells.

### Immunofluorescence

To detect RAD51 foci formation after IR treatment, B cells cultured in vitro for 48 h were exposed to 10 Gy ionizing radiation. The B cells were resuspended 4 h after IR treatment, and attached to slides using CellTak (354240; Corning). Cells were pre-extracted with ice-cold buffer consisting of 20 mM HEPES-KOH (pH 7.5), 50 mM NaCl, 3 mM MgCl2, 0.5% Triton X-100, and 300 mM sucrose. The cells were fixed in 3% formaldehyde in phosphate-buffered saline (PBS) with 2% sucrose for 10 min. After fixation, cells were permeabilized with 0.5% Triton X-100 in PBS for 10 min, followed by incubation with anti-RAD51 antibody for 1 h and subsequent rabbit Alexa 488 secondary antibody for an additional 1 h at room temperature. DAPI (80 ng/ml) was used to stain DNA and images were acquired with a Nikon Eclipse E800 microscope.

### Analytical methods and statistics

Image-based quantification of Western blots was performed with ImageJ. Full, uncropped versions of the Western blots used are included as supplemental material. Sequence alignments were performed using EMBOSS Water, using the Smith-Waterman algorithm [65]. Analysis of intrinsic disorder was by the CAID Prediction Profile [66, 67]. Statistical tests were performed as described. *P* < 0.05 was considered to be statistically significant. Where shown, n.s. indicates not significant. * indicates a *P* value < 0.05. ** indicates a *P* value < 0.01. *** indicates a *P* value < 0.001. **** indicates a *P* value < 0.0001. All experiments were conducted with a minimum of three biological replicates.

## Supplementary information

Fig. S1

Fig. S2

Fig. S3

Fig. S4

Uncropped Western blots for Fig. 2

Uncropped Western blots for Fig. S2

## Data Availability

Scans of original, uncropped western blots are provided as supplemental material. Other data is freely available from the corresponding author by request.
