# Supplementary figures and images for: Defects in DNA damage signaling and cell cycle checkpoints in a mouse model of Rhno1 deletion
Source: Cell Death Discov. 2025 Dec 19;12:54. doi: 10.1038/s41420-025-02912-z (PMC12847792; doi:10.1038/s41420-025-02912-z)

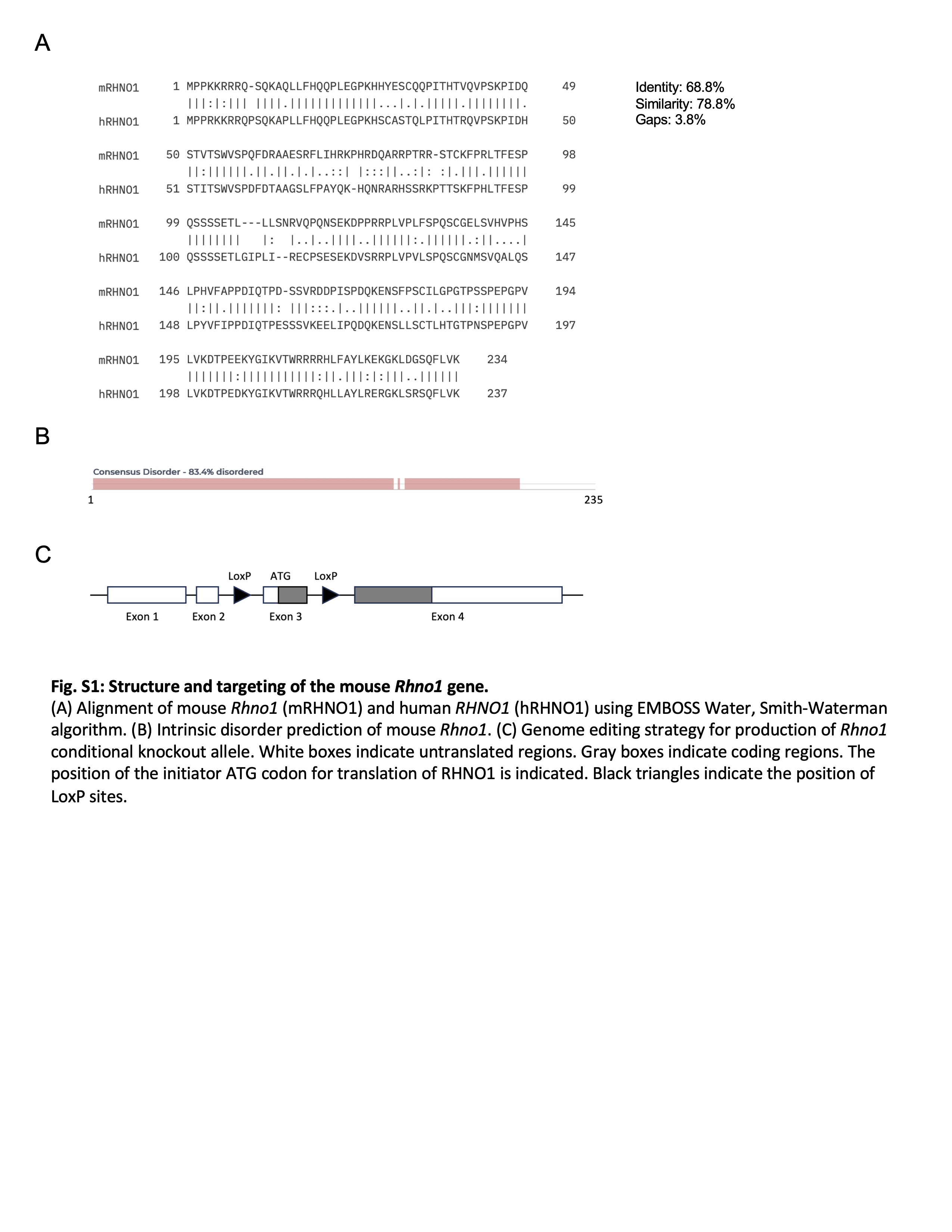

Supplement: Supplementary file 1 — Fig. S1 [file 41420_2025_2912_MOESM1_ESM.tif]

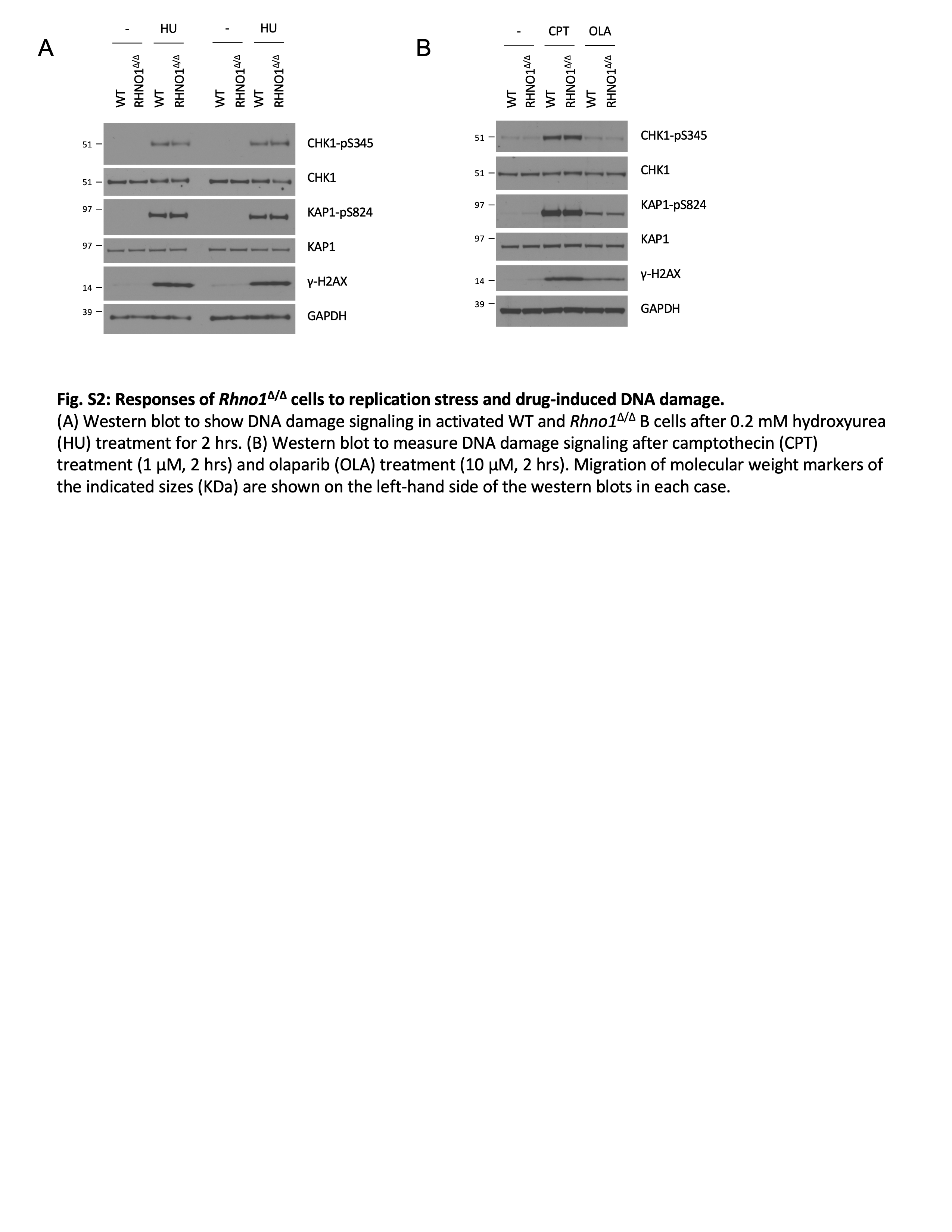

Supplement: Supplementary file 2 — Fig. S2 [file 41420_2025_2912_MOESM2_ESM.tif]

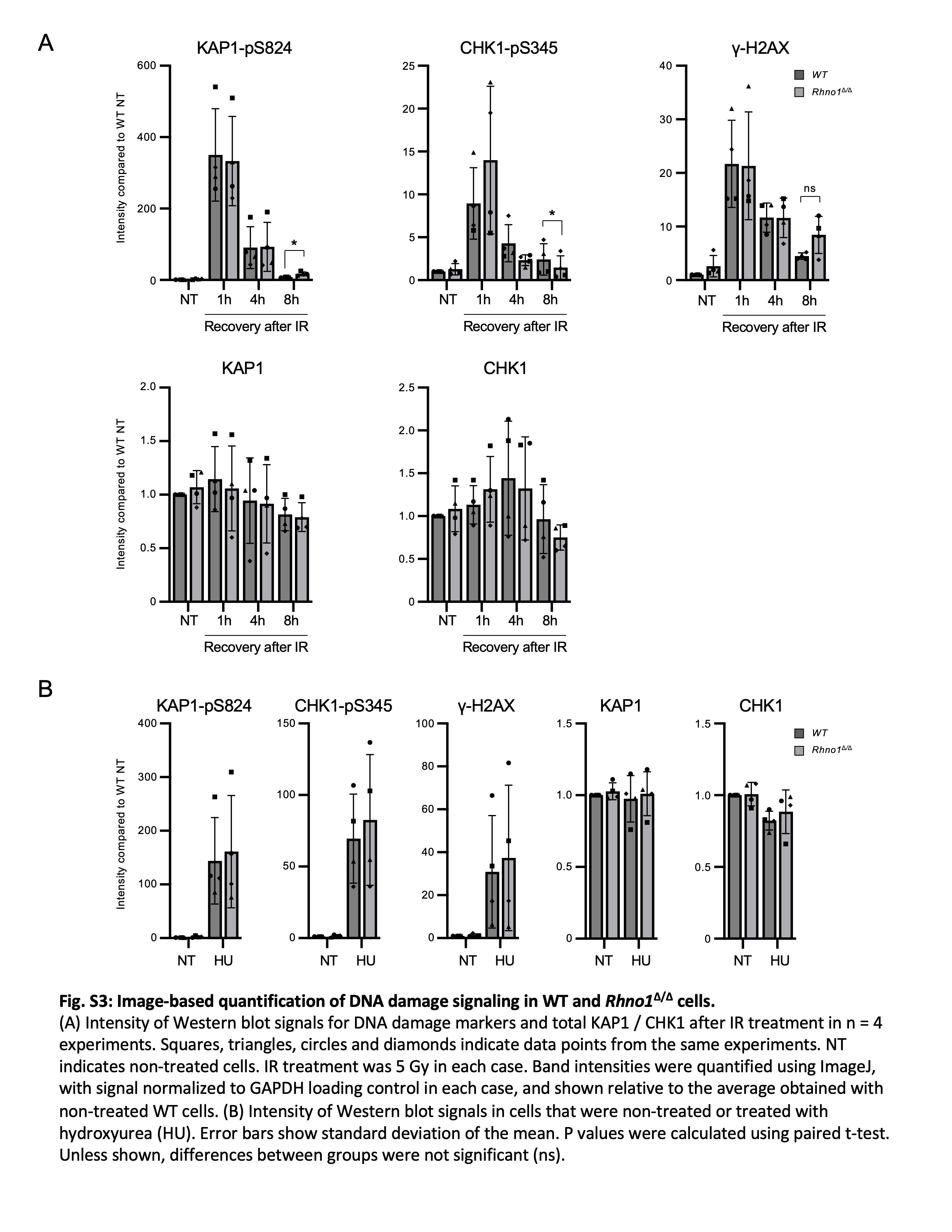

Supplement: Supplementary file 3 — Fig. S3 [file 41420_2025_2912_MOESM3_ESM.tif]

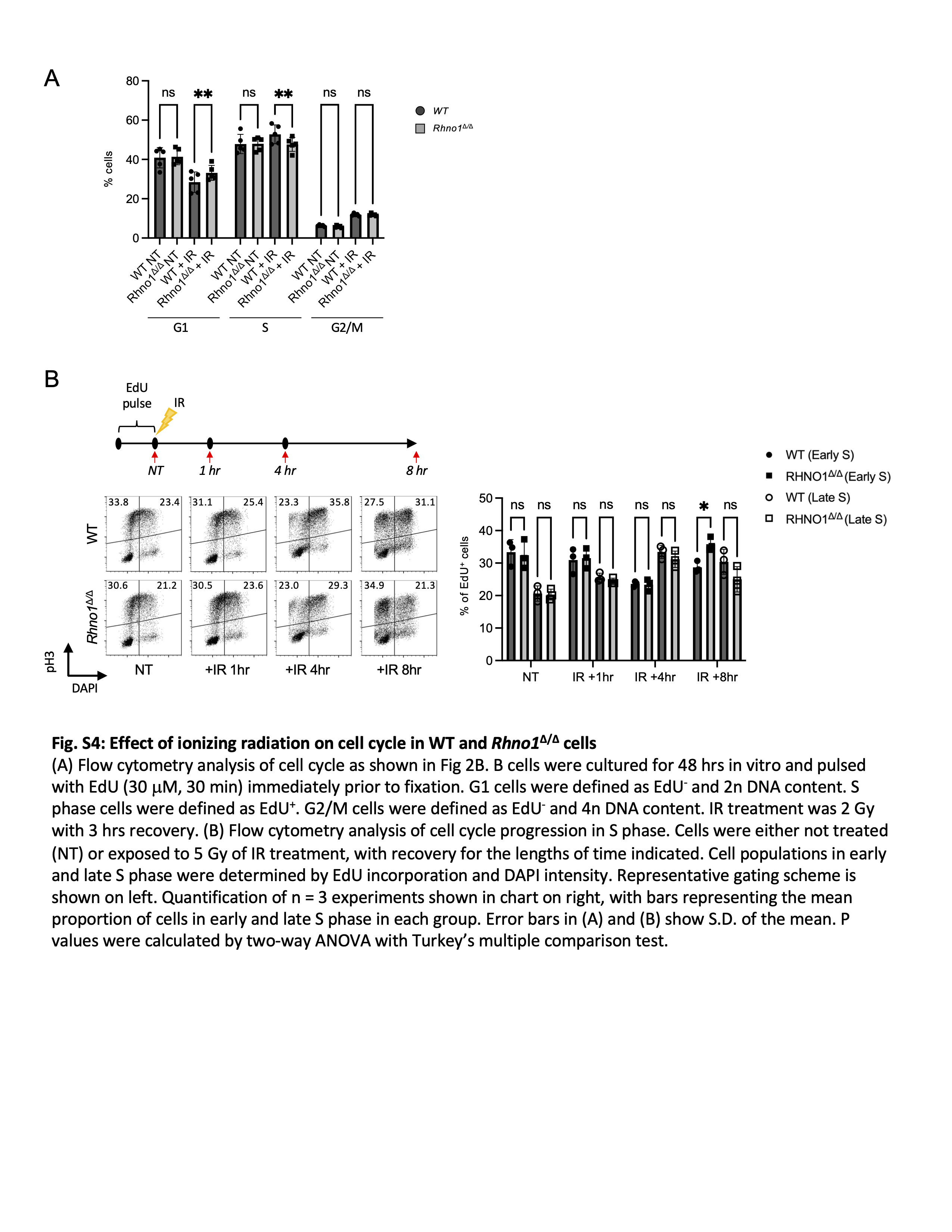

Supplement: Supplementary file 4 — Fig. S4 [file 41420_2025_2912_MOESM4_ESM.tif]

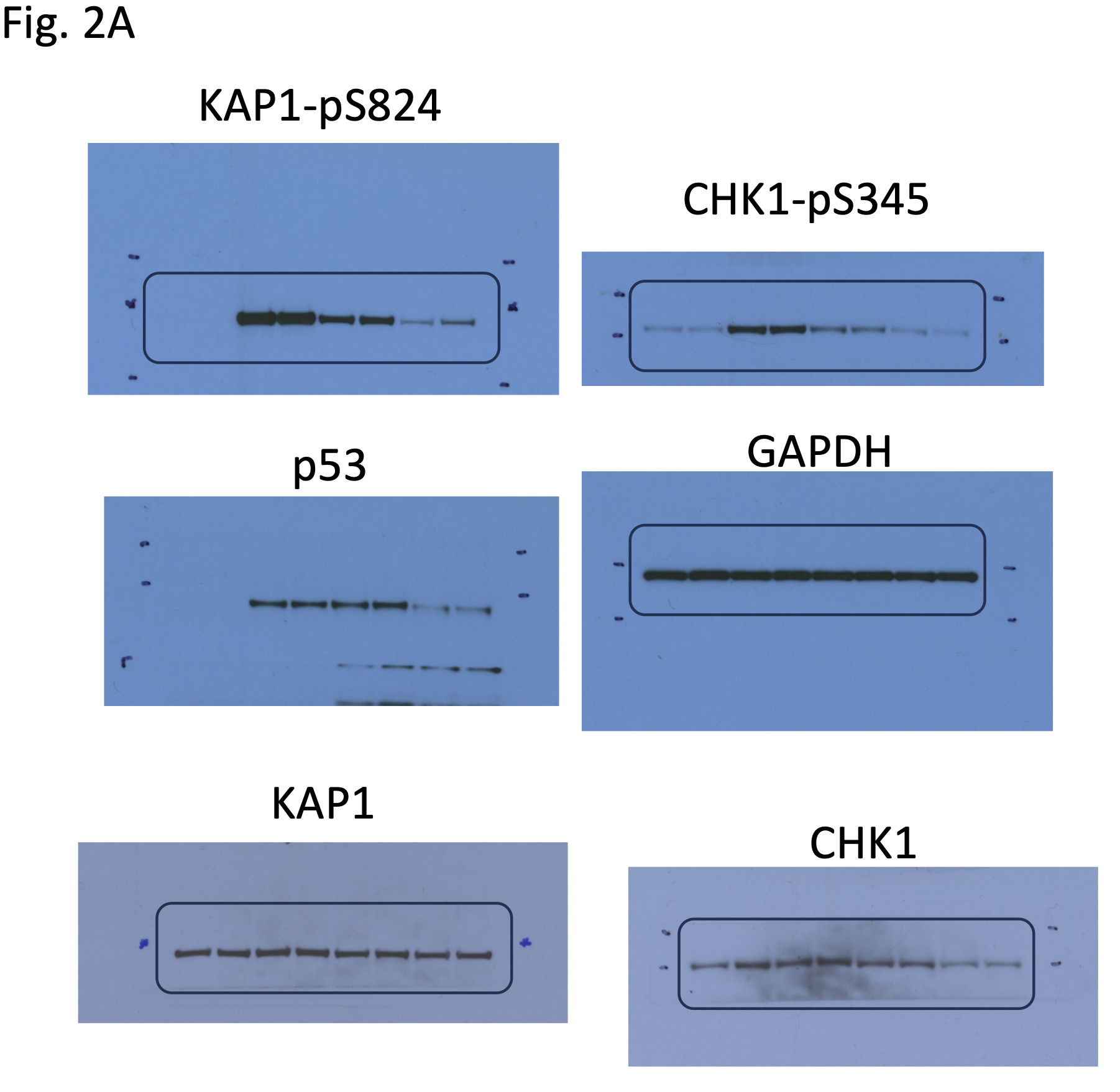

Supplement: Supplementary file 5 — Uncropped Western blots for Fig. 2 [file 41420_2025_2912_MOESM5_ESM.tif]

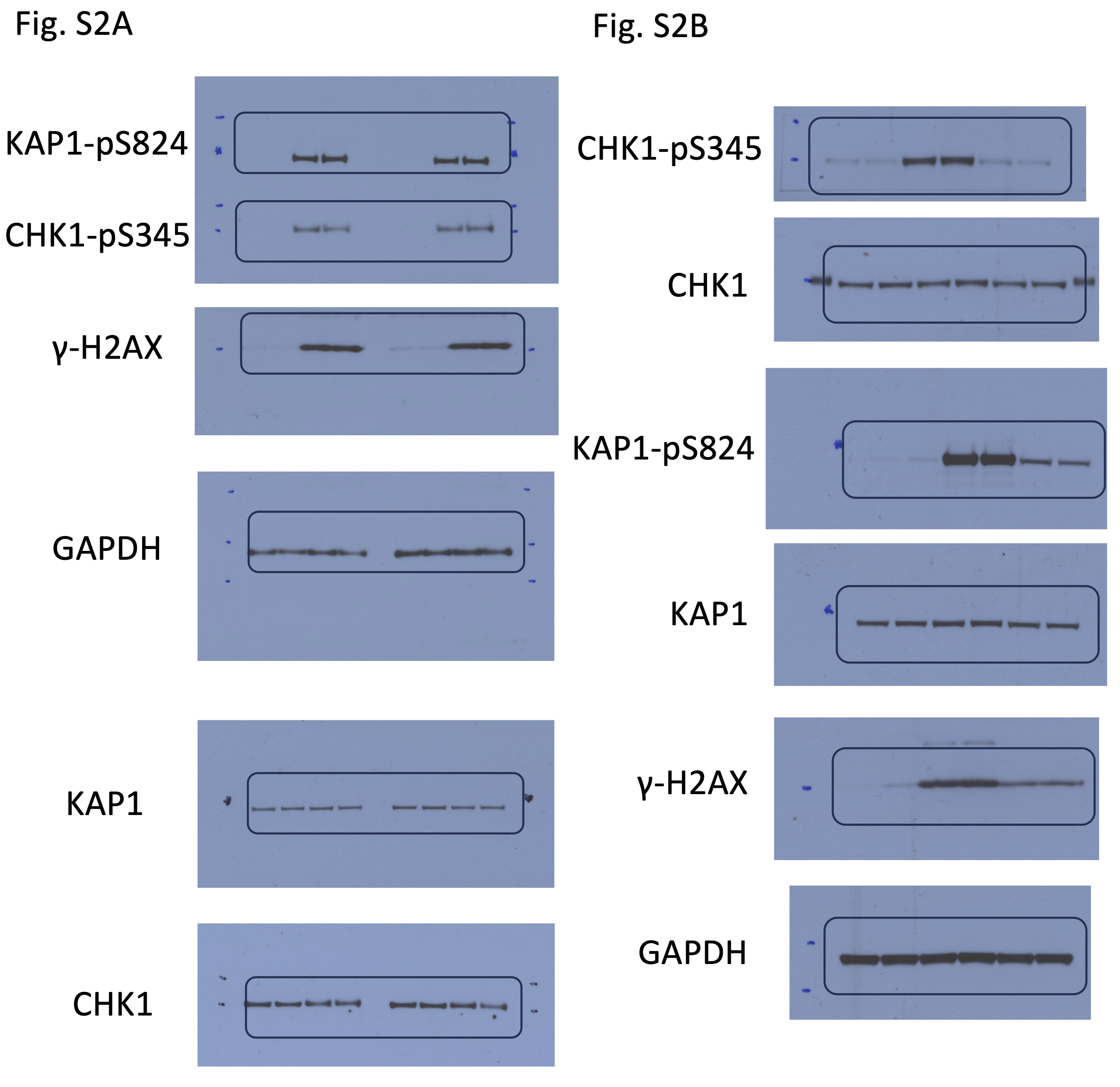

Supplement: Supplementary file 6 — Uncropped Western blots for Fig. S2 [file 41420_2025_2912_MOESM6_ESM.tif]
